# Gram-negative bacteria facilitate tumor progression through TLR4/IL-33 pathway in patients with non-small-cell lung cancer

**DOI:** 10.18632/oncotarget.24008

**Published:** 2018-01-04

**Authors:** Mengyao Sun, Yang Bai, Song Zhao, Xiyu Liu, Yongsheng Gao, Lei Wang, Bin Liu, Dashi Ma, Chunye Ma

**Affiliations:** ^1^ The Cardiac Surgery Department, The First Hospital of Jilin University, Changchun 130021, China; ^2^ The Spine Surgery Department, The First Hospital of Jilin University, Changchun 130021, China; ^3^ The Thoracic Surgery Department, Affiliated Hospital of Guilin Medical University, Guilin 541001, China

**Keywords:** gram-negative bacteria, NSCLC, TLR4, IL-33

## Abstract

Non-small-cell lung cancer (NSCLC) accounts for the most cases in clinical lung cancer patients. Patients with NSCLC are often diagnosed in advanced stage and frequently infected with gram-negative bacteria. Pulmonary infection with gram-negative bacteria is the most frequent postoperative complication in NSCLC patients. While accumulating evidence indicate an involvement of gram-negative bacteria in NSCLC progression, the underlying mechanisms remain largely unknown. Herein, we explored the effect of gram-negative bacteria on tumor progression using tumor cells from NSCLC patients. We observed that infection with gram-negative bacteria predicted advanced stages and decreased time interval to recurrence of NSCLC patients. Incubation of NSCLC cells with gram-negative bacteria promoted their growth and metastasis. Mechanistically, gram-negative bacteria activated Toll-like receptor 4 (TLR4) signaling in NSCLC cells, leading to increased mRNA and protein expression of interleukin 33 (IL-33) through MyD88-dependent pathway. Knockdown of IL-33 abrogated the contribution of gram-negative bacteria to NSCLC progression by regulating cancer metabolic activities and stem cell properties. In NSCLC patients, higher TLR4 expression was associated with increased IL-33 expression, Ki-67 proliferation index and CD133 expression in those with gram-negative bacterial infection. These findings shed new light on the molecular mechanisms underlie gram-negative bacteria mediated tumor progression and provide clues for innovative therapeutic explorations for NSCLC patients.

## INTRODUCTION

Lung cancer is the leading cause of cancer-related death worldwide [1, 2]. Non-small-cell lung cancer (NSCLC) is the dominant subtype in all clinical cases [3]. Patients with NSCLC are usually diagnosed in advanced stages, leaving the surgical operation frequently as an off-the-table option [4]. Chemotherapy and radiotherapy are the common treatments for NSCLC patients [5]. Despite significance advances in NSCLC pathogenesis and development of novel therapeutics such as targeted therapy and immunotherapy, the overall 5-year survival rate of NSCLC patients is still far less from satisfied [6–8].

If doable, surgery is the best choice for NSCLC because it provides the patients with most likelihood of cure and long-term survival [9]. Surgical operation is the primary treatment for patients with stage I and II lung cancer and an important approach in multimodality treatment for patients with stages III or with stage IV oligo-metastatic cases [10]. However, postoperative complications are critical challenges in clinical practice for NSCLC patients [11]. The most frequently observed postoperative complication is pulmonary infection that portends poor NSCLC prognosis [12]. Consequently, the long-term survival after surgery of lung cancer patients remains less than 50%, mostly due to the tumor recurrence [13].

Gram-negative bacteria are the most common pathogens in pulmonary infection of NSCLC patients, either in those with postoperative complications or in those with advanced disease, raising a possibility that gram-negative bacteria might facilitate NSCLC progression [14, 15]. Indeed, recent studies showed that gram-negative bacteria promoted the growth and metastasis of human lung cancer cell lines and primary lung cancer cells from clinical patients [16, 17]. Activation of Toll-like receptor 4 (TLR4) signaling conferred the function of gram-negative bacteria in lung cancer progression [16, 17]. However, the precise molecular mechanisms about how gram-negative bacteria affect the NSCLC progression and recurrence are still obscure.

Interleukin 33 (IL-33) is one new member of IL-1 superfamily and expressed in various tissues, especially in those with barrier functions [18, 19]. By binding to its receptor ST2, IL-33 drives production of Th2 cytokines and plays a critical role in asthma, inflammation and autoimmunity [20–23]. Recently, IL-33 in circulation was defined as a biomarker for evaluating lung cancer progression [24]. IL-33 was identified as a pro-cancer cytokine and fueled the glycolysis of NSCLC cells [25]. But whether IL-33 is involved in the crosstalk of gram-negative bacterial infection and NSCLC progression, still remains unknown.

In current study, we explored the function of gram-negative bacteria in tumor progression and recurrence in NSCLC patients. NSCLC cells isolated from surgical tissues of clinical patients would avoid the defect of established human cancer cell lines that usually do not represent the heterogeneity and characteristics of cancer cells in patients [25]. We determined an important role of IL-33 in gram-negative bacteria induced NSCLC progression and recurrence. Mechanistically, IL-33 conferred gram-negative bacteria in facilitating cancer metabolic reprograming and cancer stem cell properties. These findings could further our current knowledge in NSCLC immunopathology and provide clues for therapeutic explorations against lung cancer, especially in those with gram-negative bacterial infections.

## RESULTS

### Gram-negative bacterial infection portends advanced disease and reduced time interval to recurrence in NSCLC patients

**Figure 1:**
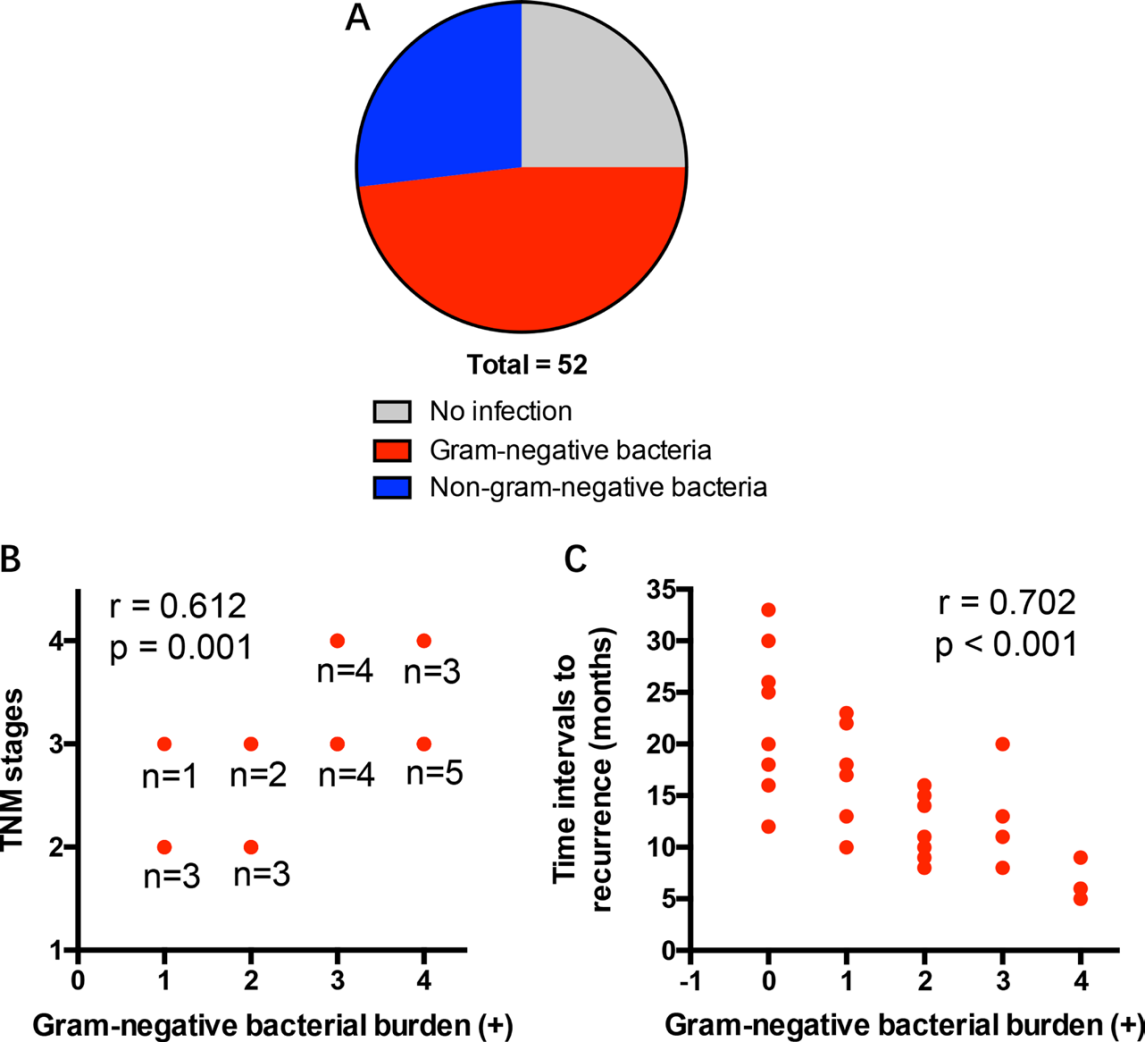
Gram-negative bacterial infection portends advanced disease and decreased tumor interval to recurrence in NSCLC patients (**A**) A cohort of 52 NSCLC patients were screened for pulmonary infection of gram-negative bacteria. (**B**) The burden of gram-negative bacteria from NSCLC patients were associated with disease progression. (**C**) The burden of gram-negative bacteria in postoperative infection were correlated with time intervals to recurrence of NSCLC patients. Each dot represents the data from one patient.

To explore the effect of pulmonary infection with gram-negative bacteria on NSCLC progression, a cohort of 52 patients diagnosed with NSCLC were detected for the presence of pulmonary gram-negative bacterial infection. While 13 patients were negative for pulmonary infection, 25 patients were positive for gram-negative bacterial infections (Figure 1A). Of interest, high burden of gram-negative bacteria predicted advanced disease stages (stage III-IV) in NSCLC patients (Figure 1B).

To evaluate the involvement of gram-negative bacteria in tumor recurrence, a cohort of 28 NSCLC patients with or without postoperative pulmonary gram-negative bacterial infection were enrolled in a follow-up study. NSCLC patients suffered from strong postoperative complication of gram-negative bacterial infection have shorter time intervals to recurrence (Figure 1C).

### Gram-negative bacteria drive outgrowth and metastasis of NSCLC through TLR4/MyD88 signaling

**Figure 2:**
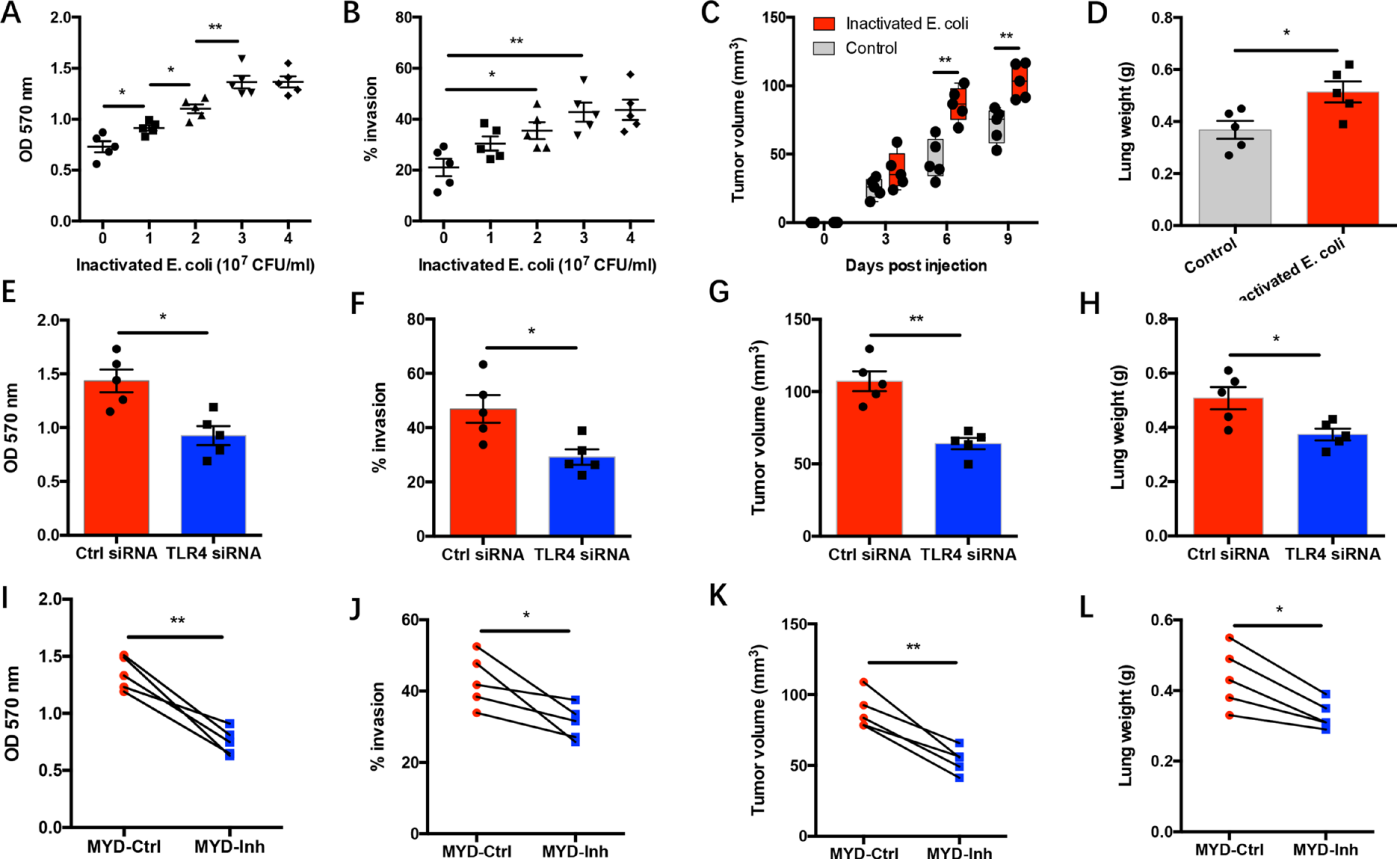
Gram-negative bacteria drive NSCLC progression via TLR4/MyD88 signaling (**A**–**B**) NSCLC cells from clinical patients (*n* = 5) were cultured with an increasing dose of heat-inactivated E. coli. Proliferative expansion of NSCLC cells was detected after 72 hours (A). Invasion of NSCLC cells was analyzed after 24 hours (B). (**C**–**D**) NSCLC cells from 5 patients were pretreated with heat-inactivated E. coli (3x10^7^ CFU/ml) for 24 hours and adoptively transferred into NOD-SCID mice. Tumor size was measured at the indicated time post NSCLC injection (C). Two weeks later, tumor metastasis was determined by analyzing lung weight to reflect tumor burden in lung (D). (**E**–**H**) NSCLC cells from 5 patients were transfected with TLR4 siRNA or control siRNA and incubated with heat-inactivated E. coli (3x10^7^ CFU/ml). NSCLC growth capacity was analyzed after 72 hours (E). NSCLC invasion was detected after 24 hours (F). (G–H) 24 hours later, NSCLC were injected into NOD-SCID mice and assayed for tumor growth on day 7 (G) and tumor metastasis on day 14 (H). (**I**–**L**) NSCLC cells from 5 patients were cultured with heat-inactivated E. coli (3x10^7^ CFU/ml) plus MyD88 inhibitory peptide (MYD-Inh, 50 μM) or control peptide (MYD-Ctrl, 50 μM). Tumor progression *in vitro* and *in vivo* were analyzed as described above. Each dot represents the data from one individual. ^*^p < 0.05. ^**^p < 0.01.

To understand the association of gram-negative bacterial infection and NSCLC progression, NSCLC cells were cultured with inactivated gram-negative bacteria [16, 17]. As expected, gram-negative bacteria promoted the growth capacity and invasion of NSCLC cells (Figure 2A–2B). Gram-negative bacteria enhanced tumor progression in a dose dependent manner and doubled the proliferative expansion rate and the number of invading NSCLC cells. The *in vivo* conformation was performed with NOD-SCID mice challenged with NSCLC cells that were pretreated with gram-negative bacteria. Prior treatment with gram-negative bacteria promoted the growth and metastasis of NSCLC cells in immune-deficient mice (Figure 2C–2D). Further, genetic knockdown of TLR4 expression in NSCLC cells efficiently abrogated the gram-negative bacteria mediated tumor progression both *in vitro* and *in vivo* (Supplementary Figure 1, Figure 2E–2H). These findings are consistent with previous studies [16, 17], pinpointing the requirement of TLR4 receptor in gram-negative bacteria mediated lung cancer progression. Accordingly, blocking MyD88 signaling by administration of MyD88 inhibitory peptide significantly inhibited gram-negative bacteria mediated NSCLC progression (Figure 2I–2L).

### TLR4 activation by gram-negative bacteria induces NSCLC progression in IL-33 dependent manner

**Figure 3:**
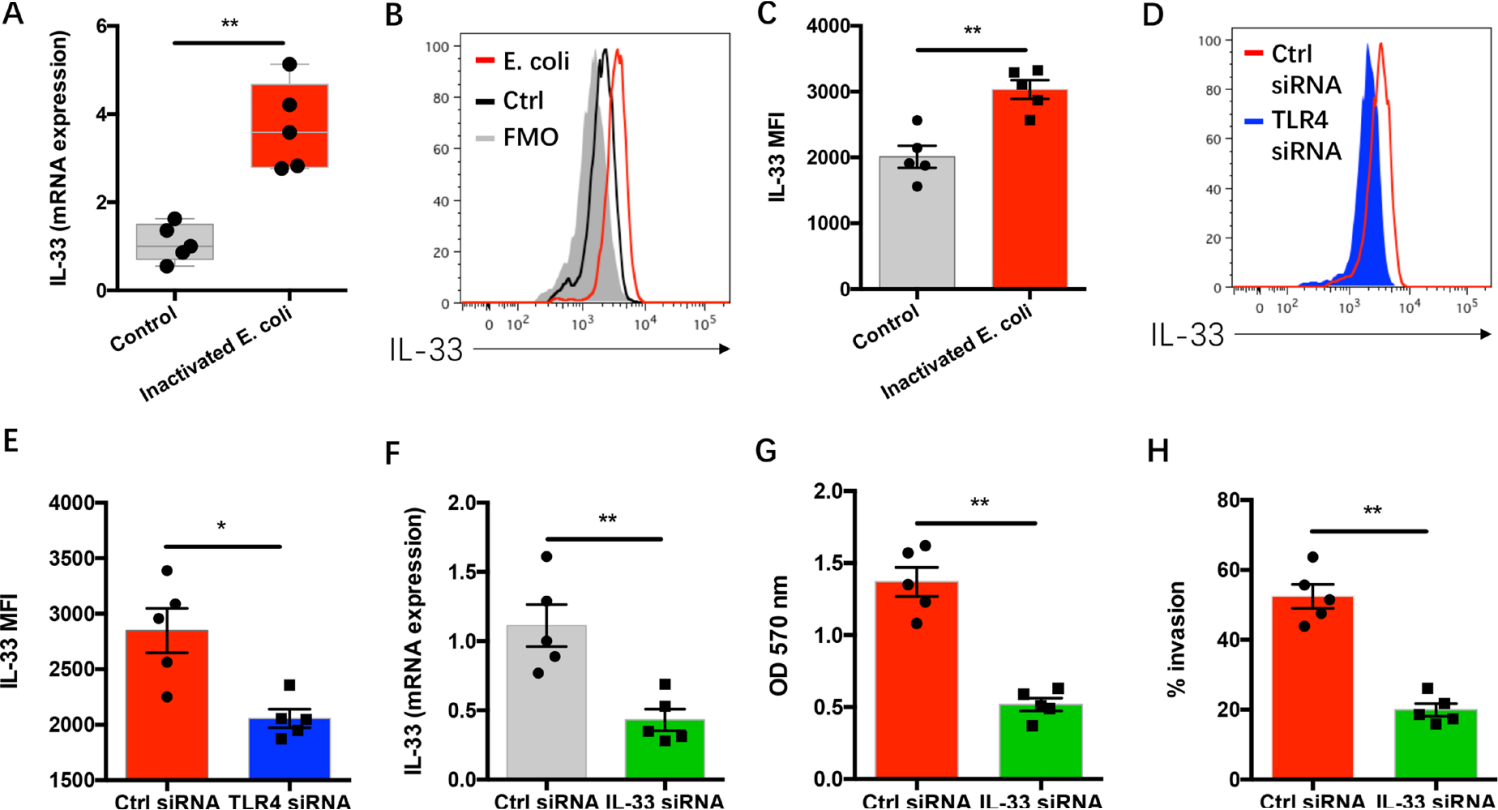
Gram-negative bacteria-induced NSCLC progression relies on TLR4/IL-33 pathway (**A**) NSCLC cells from 5 patients were incubated with or without heat-inactivated E. coli (3x10^7^ CFU/ml) for 12 hours and analyzed for IL-33 mRNA expressions. (**B**–**C**) NSCLC cells from clinical patients (*n* = 5) were incubated with or without heat-inactivated E. coli (3x10^7^ CFU/ml) for 24 hours and analyzed for IL-33 protein expressions. (**D**–**E**) NSCLC cells from 5 clinical patients were transfected with TLR4 siRNA or control siRNA and incubated with heat-inactivated E. coli (3x10^7^ CFU/ml) for 24 hours. IL-33 protein expressions were detected by flow cytometry. (**F**) NSCLC cells from 5 patients were transfected with IL-33 siRNA or control siRNA and analyzed for IL-33 mRNA expressions after 12 hours. (**G**–**H**) NSCLC cells from 5 patients were transfected with IL-33 siRNA or control siRNA and incubated with heat-inactivated E. coli (3x10^7^ CFU/ml). NSCLC growth capacity was detected after 72 hours (G) and the invasion was determined after 24 hours (H). Each dot represents the data from one patient. ^*^p < 0.05. ^**^p < 0.01.

To detect whether IL-33 was involved in the effect of gram-negative bacteria on NSCLC progression, NSCLC cells were incubated with inactivated gram-negative bacteria and analyzed for IL-33 expressions. We found that gram-negative bacteria efficiently induced mRNA and protein expressions of IL-33 in NSCLC cells (Figure 3A–3C). Genetic knockdown of TLR4 expression significantly reduced IL-33 expression in response to gram-negative bacteria (Figure 3D–3E).

To evaluate the potential role of IL-33 in gram-negative bacteria mediated NSCLC progression, NSCLC cells were transfected with IL-33 siRNA and cultured with inactivated gram-negative bacteria. Knockdown of IL-33 expression abrogated gram-negative bacteria mediated NSCLC progression (Supplementary Figure 2 and Figure 3F–3H).

### IL-33 confers gram-negative bacteria-enhanced cancer metabolism

**Figure 4:**
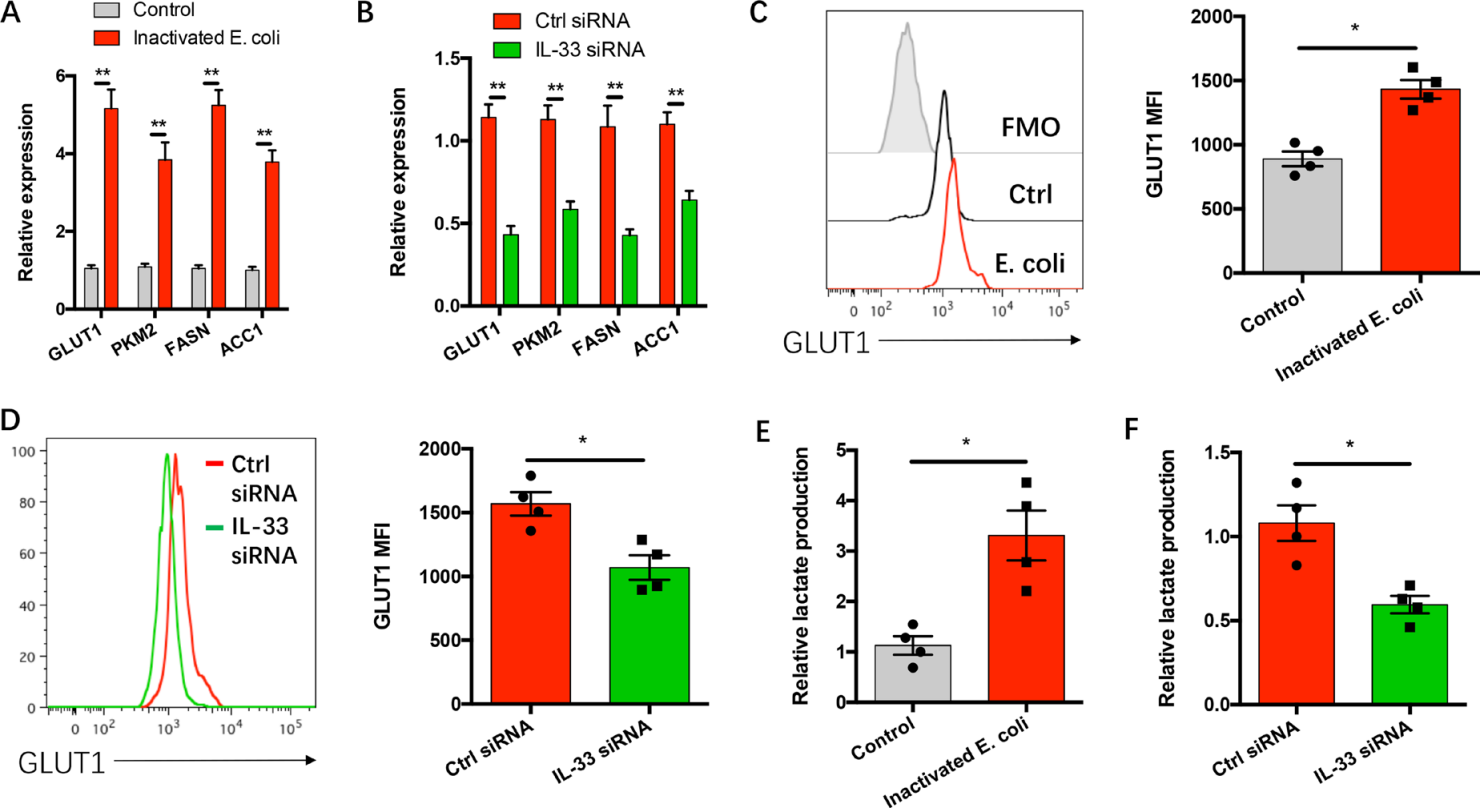
IL-33 confers gram-negative bacteria-induced cancer metabolism (**A**) NSCLC cells from 5 clinical patients were incubated with or without heat-inactivated E. coli (3x10^7^ CFU/ml) for 12 hours and detected the mRNA expressions of the indicated genes. (**B**) NSCLC cells from 5 clinical patients were transfected with IL-33 siRNA or the control siRNA, and incubated with heat-inactivated E. coli (3x10^7^ CFU/ml) for 12 hours. (**C**, **E**) NSCLC cells from 4 clinical patients were incubated with or without heat-inactivated E. coli (3x10^7^ CFU/ml) for 36 hours. (**D**, **F**) NSCLC cells from 4 clinical patients were transfected with IL-33 siRNA or the control siRNA, and incubated with heat-inactivated E. coli (3x10^7^ CFU/ml) for 36 hours. Each dot represents the data from one patient. ^*^p < 0.05. ^**^p < 0.01.

High rates of glycolysis and lipogenesis are two hallmarks of cancer metabolic reprograming to support their uncontrolled outgrowth and metastasis [16, 25, 26]. To explore how IL-33 regulates gram-negative bacteria induced NSCLC progression, NSCLC cells with or without IL-33 knockdown were incubated with inactivated gram-negative bacteria and analyzed for expressions of cancer metabolism reflecting genes GLUT1, PKM2, FASN and ACC1. While gram-negative bacteria increased their mRNA expressions (Figure 4A), this process could be abrogated by IL-33 knockdown (Figure 4B). IL-33 is essential for gram-negative bacteria to enhance the membrane GLUT1 protein levels (Figure 4C–4D). Accordingly, gram-negative bacteria promoted lactate production in NSCLC cells, which again could be impeded by IL-33 knockdown (Figure 4E–4F).

### IL-33 licenses gram-negative bacteria-induced cancer stem cell properties

**Figure 5:**
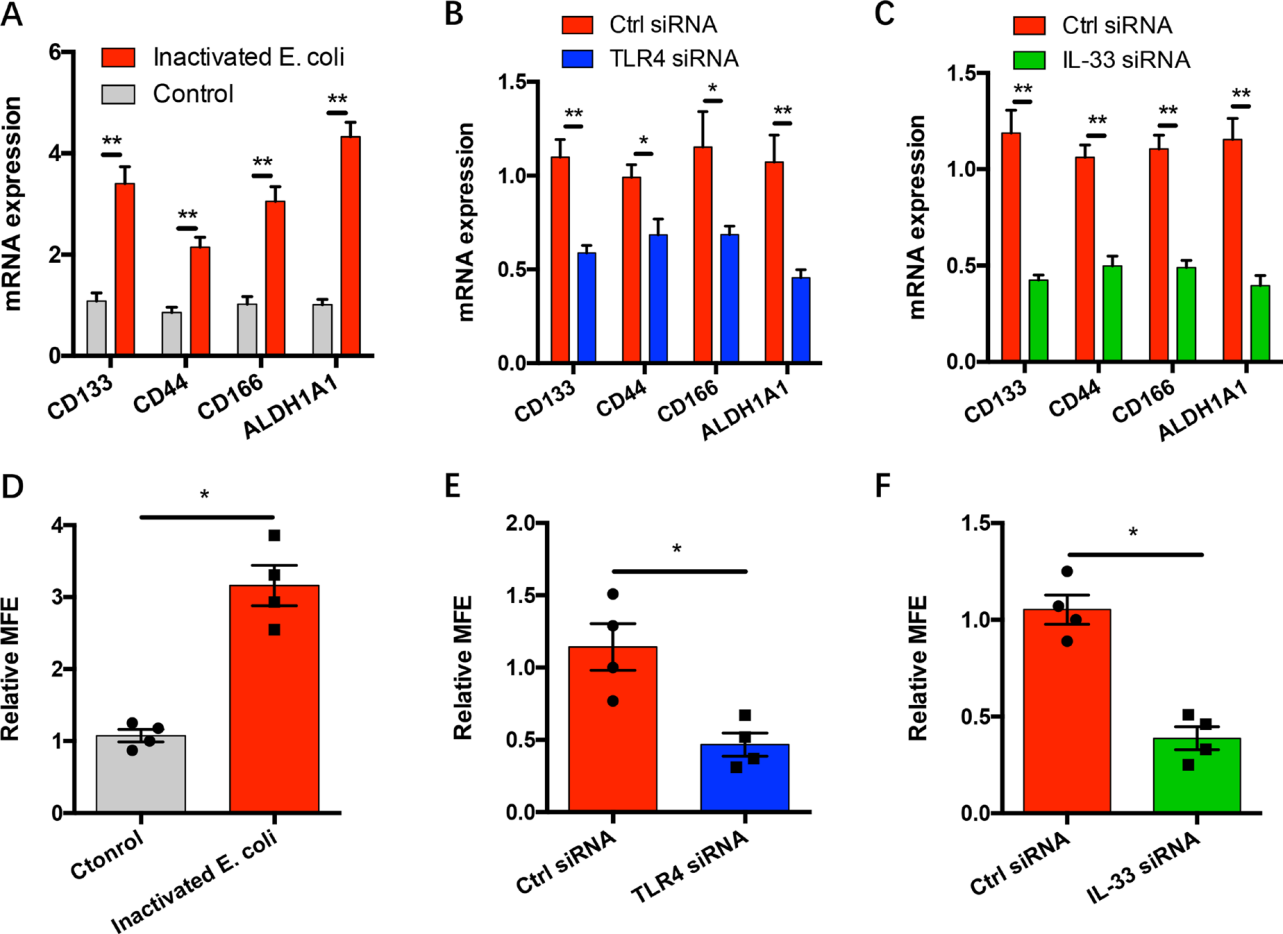
IL-33 licenses gram-negative bacteria-induced stem cell properties (**A**) NSCLC cells from 5 patients were incubated with heat-inactivated E. coli (3x10^7^ CFU/ml) for 12 hours and detected for mRNA expressions of the indicated CSC-related genes. (**B**–**C**) NSCLC cells from 5 patients were transfected with TLR4 siRNA, IL-33 siRNA or the control respectively, incubated with heat-inactivated E. coli (3x10^7^ CFU/ml) for 12 hours. (**D**) NSCLC cells from 4 patients were incubated with or without heat-inactivated E. coli (3x10^7^ CFU/ml) and detected for MFE. (**E**–**F**) NSCLC cells from 4 patients were transfected with TLR4 siRNA, IL-33 siRNA or the control respectively, incubated with heat-inactivated E. coli (3x10^7^ CFU/ml) and analyzed for MFE. Each dot represents the data from one patient. ^*^p < 0.05. ^**^p < 0.01.

Cancer stem cells (CSCs) are well-acknowledged as a niche of cancer cells that are endowed with stem cell properties, chemotherapy resistant, hardly eradicated by clinical management and thus lead to tumor recurrence [27, 28]. Incubation with gram-negative bacteria increased the expressions of CSC-related genes including CD133, CD44, CD166 and ALDH1A1 (Figure 5A). This process was dependent on TLR4 signaling and IL-33 expression in NSCLC cells and could be abrogated by knockdown of TLR4 and IL-33 expressions (Figure 5B–5C). In support, gram-negative bacteria significantly enhanced the mammosphere formation through TLR4 activation and IL-33 upregulation in NSCLC cells (Figure 5D–5F).

### TLR4/IL-33 pathway correlates with tumor growth and expression of CSC-related gene in NSCLC patients with gram-negative bacterial infection

**Figure 6:**
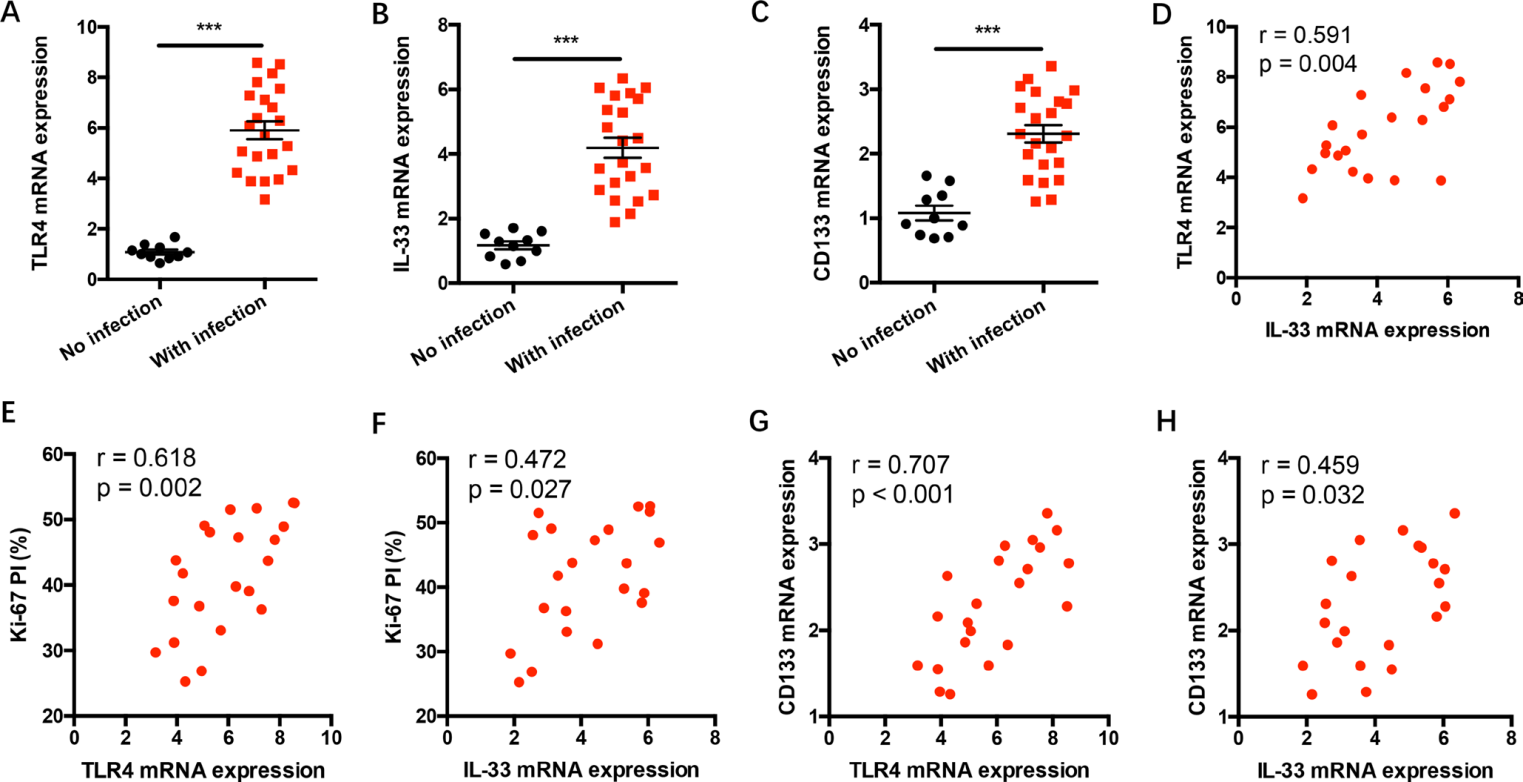
TLR4/IL-33 signaling correlates with tumor progression in NSCLC patients with gram-negative bacterial infection (**A**–**C**) NSCLC patients with or without gram-negative bacterial infection were detected for mRNA expressions of TLR4, IL-33 and CD133 in tumor tissues. (**D**–**H**) TLR4 and IL-33 expressions were analyzed for correlations with tumor Ki-67 proliferation index and CSC-related gene CD133 expression in gram-negative bacteria affected NSCLC tissues. Each dot represents the data from one patient. ^***^p < 0.001.

To investigate the *in vivo* relevance of TLR4 signaling with IL-33 expression and NSCLC progression in clinical patients, NSCLC tissues with or without gram-negative bacterial infection were analyzed for TLR4, IL-33 and CD133 expressions. We observed higher TLR4, IL-33 and CD133 expressions in NSCLC patients with gram-negative bacterial infection (Figure 6A–6C). TLR4 expressions were closely correlated with IL-33 expressions in gram-negative bacterial infected patients (Figure 6D). Of note, TLR4 and IL-33 expressions were positively associated with Ki-67 proliferative index (PI) and expressions of CSC-related gene CD133 in NSCLC patients with gram-negative bacterial infection (Figure 6E–6H).

## DISCUSSION

The relationship between pathogenic infection and cancer progression, especially the pulmonary infection and lung cancer, has long been a subject of active investigation [16, 17]. Gram-negative bacterial infection is the most common pathogen that affects patients with NSCLC, either in patients with postoperative complications or in patients with advanced disease [16, 17, 29]. Herein, we identified a novel mechanism underlies gram-negative bacteria mediated NSCLC progression. Specifically, by stimulating TLR4 signaling, gram-negative bacteria induced robust IL-33 expression in NSCLC cells, facilitating cancer metabolic reprograming and development of cancer stem cell properties.

Toll-like receptors are pattern recognition receptors that recognize pathogen-associated molecular patterns, a crucial protective mechanism in innate immunity and human diseases [30, 31]. Accumulating evidence have demonstrated an important function of TLR signaling in lung cancer pathogenesis [32, 33]. As such, activation of TLR4 or TLR9 enhanced growth and metastasis of lung cancer [34–37]. Triggering of TLR7 and TLR8 expressed by human lung cancer cells induced cell survival and chemo-resistance [38]. In current study, we determined that blockade of TLR4 signaling efficiently abrogated gram-negative bacteria mediated NSCLC progression. In consistent, gram-negative bacteria activated TLR4 in NSCLC cells, promoting tumor growth and metastasis [16, 17]. It is reasonable because lipopolysaccharides, the major component of the outer membrane of gram-negative bacteria, is a well-known ligand for TLR4 [35, 39].

IL-33 is recognized as a dual-functional factor in maintenance of barrier functions and in driving Th2 response [19, 23]. Recent studies identified IL-33 as a critical factor involved in cancer pathogenies [40, 41]. IL-33 signaling contributed to intestinal tumorigenesis in humans and mice [42]. IL-33/ST2 pathway facilitated metastasis of human colorectal cancer [43]. IL-33 promoted growth and liver metastasis of colorectal cancer in mice by remodeling the tumor microenvironment and inducing angiogenesis [44]. Besides, IL33/ST2 axis could promote epithelial cell transformation and breast tumorigenesis via upregulation of COT activity [45]. IL-33/ST2 axis promoted breast cancer growth and metastases by facilitating intratumoral accumulation of immunosuppressive and innate lymphoid cells [46]. In addition, IL-33 expression predicted poor prognosis and promoted ovarian cancer cell growth and metastasis through regulating ERK and JNK signaling pathways [47]. IL-33 also promoted gastric cancer cell metastasis through ST2-ERK1/2 pathway [48]. Similarly, higher decline extent of serum IL-33 level was a predictor of shorter progression-free survival after chemotherapy in gastric cancer patients [49]. Mechanistically, IL-33 activated tumor stroma to promote intestinal polyposis [50]. IL-33 in tumor microenvironment was crucial for the accumulation and function of myeloid-derived suppressor cells [51]. Herein, we extended previous studies by demonstrating that gram-negative bacterial infection led to high expression of IL-33 in NSCLC cells through TLR4 activation. IL-33 knockdown decreased the enhanced cancer glycolysis, lipogenesis and stemness by gram-negative bacteria, suggesting IL-33 as an effective regulator of cancer metabolism and cancer stem cell properties. In consistent with these findings, IL-33 signaling promoted membrane GLUT1 localization and glycolysis of NSCLC cells [25]. IL-33 facilitated endocrine resistance of breast cancer by inducing cancer stem cell properties [52]. Of important, blockade of IL-33 restricted tumor progression of human NSCLC xenograft in immune-deficient mice [53].

IL-33 production could be induced by TLR agonists in IRF3 dependent manner [54]. TLR4 activation upregulated IL-33 expression in bone marrow-derived dendritic cells, alveolar macrophages, and respiratory dendritic cells [55]. TLR4 activation by short ragweed pollen led to IL-33 mRNA and protein expression in MyD88 and NF-kB dependent manner in ocular surface, cervical lymph nodes and isolated CD4 T cells of BALB/c mice [56]. Cyclic stretch activated TLR4 signaling to induce IL-33 production through HMGB1 in murine respiratory epithelial cells [57]. On the other hand, IL-33 increased the expression of the LPS receptor components MD2 and TLR4, the soluble form of CD14 and the MyD88 adaptor molecule, enhancing the LPS-induced pro-inflammatory response in macrophage [58]. Combing these findings indicate a positive feedback loop for TLR4/IL-33 pathway. Herein, we showed that gram-negative bacteria stimulation resulted in up-regulation of IL-33 expression in NSCLC cells and this process could be inhibited by TLR4 knockdown. Considering the aberrant expression of IRF3 in human lung cancer and the crucial role of NF-kB activity in lung cancer carcinogenesis [59, 60], the mechanisms underlie TLR4-induced IL-33 expression in NSCLC cells might involve IRF3 and NF-kB pathway, as well as HMGB1 since it binds to LPS to facilitate LPS transfer and initiate TLR4 signaling [61].

It should be pointed out that plasma IL-33 levels could be inversely associated with progression of lung cancer [62]. IL-33 was reported to enhance programmed oncosis of ST2L-positive low-metastatic Lewis lung carcinoma [63]. Tumoral expression of IL-33 could inhibit tumor growth and modify tumor microenvironment through CD8 T and NK cells in mice bearing B16 cells and 4T1 cells [64]. IL-33 could restrict tumor growth and inhibit pulmonary metastasis in melanoma-bearing mice through eosinophils [65]. Systemic administration of recombinant IL-33 protein could inhibit growth of established tumors in transplant and de novo melanoma tumorigenesis models through inducing CD8 T cell expansion and IFN-γ production, as well as through restoring dendritic cell activation and maturation [66]. These studies suggest an anti-cancer activity of IL-33 in tumor immunity, reflecting a complexity of IL-33 in cancer pathogenesis. These conflicting phenomena might due to distinct types of cancer and different cohorts of clinical patients. Clinical therapeutics such as radiotherapy and chemotherapy could induce IL-33 expressions in tumor tissue of clinical patients [67, 68], suggesting clinical managements as critical factors in determining IL-33 expressions. Besides, research findings from established cancer cell lines could be different with those from primary cancer cells in clinical patients. We propose tumoral IL-33 expression and its function in tumor progression as an individualized element to be considered in lung cancer patients.

In essence, pulmonary infection with gram-negative bacteria in NSCLC patients results in TLR4 activation and IL-33 overexpression, promoting tumor progression and recurrence by enhancing cancer metabolic reprograming and cancer stem cell properties. These findings derived from primary tumor cells reflect a realistic condition in clinical patients. Blocking TLR4/IL-33 pathway could be an optimistic strategy in controlling NSCLC progression and recurrence.

## MATERIALS AND METHODS

### Patients

A cohort of 121 patients diagnosed with NSCLC was enrolled in this study. Surgical tissues and clinical parameters were collected after written consent form was obtained. The NSCLC patients were forty-one to eighty-three years old. Fifty-eight patients were males and eighty-one cases were adenocarcinoma. The burden of infected gram-negative bacteria was from medical laboratory. Ki-67 proliferation index was from pathology division. Experiments were performed in accordance with the 1964 Helsinki declaration including its later amendments and approved by Jilin Institutional Ethics Committee.

### Mice

Immuno-deficient NOD-SCID mice of 8–10 weeks old were from Beijing Vital River Laboratory Animal Technology Co., Ltd and housed under specific pathogen free animal facility. Experiments were approved by Jilin Institutional Ethics Committee.

### Cells and reagents

Clinical surgical tissues were dissociated into a single-cell suspension with the human Tumor Dissociation Kit (Miltenyi Biotec). NSCLC cells were isolated using the human Tumor Cell Isolation Kit (Miltenyi Biotec). Experiments were performed according to the manufactures’ instructions. Cells were cultured in complete RPMI 1640 medium containing 10% FBS (Thermo Fisher Scientific) plus Penicillin-Streptomycin-Glutamine (1:100, Thermo Fisher Scientific) at 37°C under 5% CO2 as previously described [25].

Heat-inactivation of gram-negative bacteria Escherichia coli (ATCC^®^ 11775™) was performed as previously described [16, 17]. Human TLR4 siRNA, IL-33 siRNA and the controls were from Santa Cruz Biotechnology. MyD88 inhibitory peptide and control peptide were from *In vivo* Gen. Cellular Nucleofector™ kit was from Lonza. All reagents were used according to the manufactures’ instructions.

### Real-time PCR

Real-time qPCR was performed using SYBR Green qPCR Master Mix (Bimake) as previously described [69]. All qPCR primers were from Sino Biological. Beta-actin was used as the internal control.

### Flow cytometry

Flow cytometric analysis with NSCLC cells were performed as previously described [25, 69]. Briefly, 0.2 million NSCLC cells were fixed with BD Fix Buffer I, washed and stained with APC labeled anti-human TLR4 antibody (BioLegend) or PE anti-human GLUT1 antibody (R&D Systems) at 4°C for 45 min. Otherwise, cells were fixed with BD Fix Buffer I, permeabilized with BD Perm Buffer III, washed and stained with PE labeled anti-human IL-33 antibody (R&D Systems) for 45 min under room temperature. Cells were acquired using BD LSR II flow cytometer and data analysis was performed with FlowJo software (Tree Star).

### Lactate production

Lactate production was determined with Lactate Assay Kit (Sigma) as previously described [25].

### Mammosphere (MS) formation assay

NSCLC cells were seeded at 1×10^5^ cells/ml at ultralow attachment plates and cultured for 7 days. MS with a diameter ≥ 50μm were counted and MS-forming efficiency (MFE) was calculated by dividing the number of MS with original number of seeded cells [52].

### MTT assay

*In vitro* growth capacity of NSCLC cells was determined with MTT assay [16, 25]. Briefly, 10 thousand cells were incubated with or without heat-inactivated E. coli for 72 hours and detected for proliferative expansion with MTT cell proliferation kit (Cayman Chemical) according to the manufacturer’s instructions.

### Invasion assay

*In vitro* invasion of NSCLC cells was detected using Corning^®^ BioCoat™ Matrigel^®^ Invasion Chambers [16, 25]. 0.1 million NSCLC cells suspended in 0.5 mL of serum-free culture medium were seeded onto the upper chamber of the Matrigel inserts. Complete culture medium containing 10% FBS was placed into the lower chambers. 24 hours later, invading cells on the lower side of the chamber were fixed, stained and counted under microscope. Data is expressed as the percent invasion through the Matrigel inserts relative to the control inserts.

### Tumor growth

Tumor growth *in vivo* was performed in NOD-SCID mice as previously described [16, 25, 34, 35]. Briefly, 5 million NSCLC cells were pretreated with or without heat-inactivated E. coli for 24 hours and subcutaneously injected into NOD-SCID mice. Tumor growth was determined by measuring the tumor size with digital calipers. NSCLC cells that failed in tumorigenesis were excluded from the study.

### Tumor metastasis

Tumor metastasis *in vivo* was performed in NOD-SCID mice according to previously described method [25, 70–72]. NOD-SCID mice were intratracheally challenged with LPS (10 μg/mouse, Sigma) and injected with 1 million NSCLC cells through the tail veins after 6 hours. Tumor metastasis was determined by lung weight to reflect the tumor burden at 14^th^ day post NSCLC injection.

### Statistical analyses

Data were presented as mean ± SEM. Non-parametric Mann-Whitney *U* test and Spearman rank correlation coefficient were applied for statistical analysis using GraphPad Prism 6.0 software. *P* < 0.05 was considered as statistically significant.

## SUPPLEMENTARY MATERIALS FIGURES



## Abbreviations

NSCLC: non-small-cell lung cancer
TLR4: Toll-like receptor 4
MyD88: myeloid differentiation primary-response protein 88
IL-33: interleukin-33
