# Supplementary material for: Gram-negative bacteria facilitate tumor progression through TLR4/IL-33 pathway in patients with non-small-cell lung cancer
Source: Oncotarget. 2018 Jan 4;9(17):13462–73. doi: 10.18632/oncotarget.24008 (PMC5862591; doi:10.18632/oncotarget.24008)
Supplement: Supplementary file 1 [file oncotarget-09-13462-s001.pdf]

# Gram-negative bacteria facilitate tumor progression through TLR4/IL-33 pathway in patients with non-small-cell lung cancer

## SUPPLEMENTARY MATERIALS

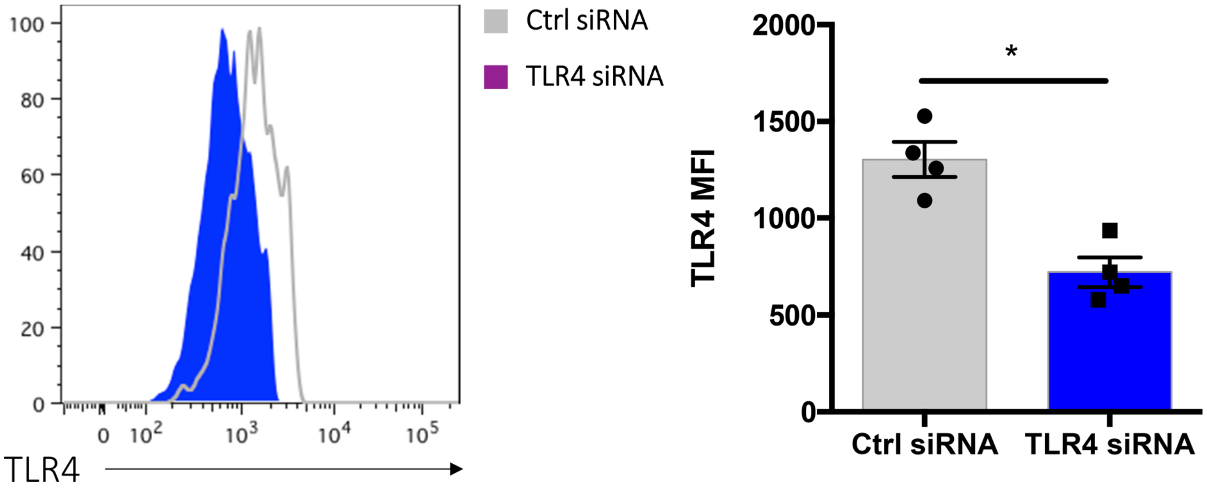


**Supplementary Figure 1: Knockdown of TLR4 expression in NSCLC cells.** NSCLC cells from 4 patients were transfected with TLR4 siRNA or control siRNA and detected for TLR4 protein expression by flow cytometry after 24 hours. Representative and collective MFI were shown. Each dot represents the data from one patient. $^{*}p < 0.05$.

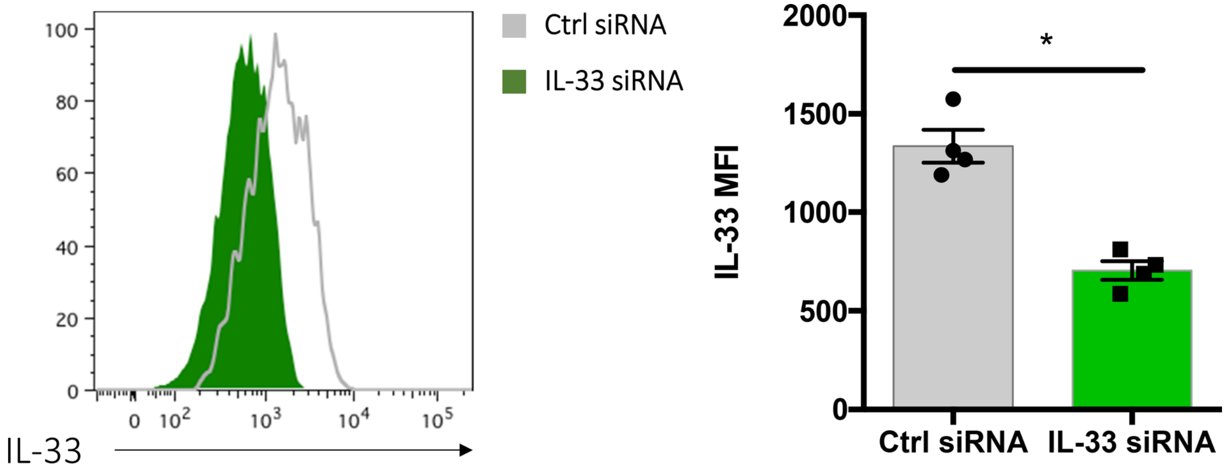


**Supplementary Figure 2: Knockdown of IL-33 expression in NSCLC cells.** NSCLC cells from 4 patients were transfected with IL-33 siRNA or control siRNA and detected for IL-33 protein expression by flow cytometry after 24 hours. Representative and collective MFI were shown. Each dot represents the data from one patient. $^{*}p < 0.05$.
